# Transcriptome analysis of ripe and unripe fruit tissue of banana identifies major metabolic networks involved in fruit ripening process

**DOI:** 10.1186/s12870-014-0316-1

**Published:** 2014-12-02

**Authors:** Mehar Hasan Asif, Deepika Lakhwani, Sumya Pathak, Parul Gupta, Sumit K Bag, Pravendra Nath, Prabodh Kumar Trivedi

**Affiliations:** CSIR-National Botanical Research Institute, Council of Scientific and Industrial Research (CSIR-NBRI), Rana Pratap Marg, Lucknow, 226001 India; Academy of Scientific and Innovative Research (AcSIR), Anusandhan Bhawan, 2 Rafi Marg, New Delhi, 110 001 India

**Keywords:** Banana, Ethylene, Fruit ripening, *Musa acuminata*, Transcriptome

## Abstract

**Background:**

Banana is one of the most important crop plants grown in the tropics and sub-tropics. It is a climacteric fruit and undergoes ethylene dependent ripening. Once ripening is initiated, it proceeds at a fast rate making postharvest life short, which can result in heavy economic losses. During the fruit ripening process a number of physiological and biochemical changes take place and thousands of genes from various metabolic pathways are recruited to produce a ripe and edible fruit. To better understand the underlying mechanism of ripening, we undertook a study to evaluate global changes in the transcriptome of the fruit during the ripening process.

**Results:**

We sequenced the transcriptomes of the unripe and ripe stages of banana (*Musa accuminata*; Dwarf Cavendish) fruit. The transcriptomes were sequenced using a 454 GSFLX-Titanium platform that resulted in more than 7,00,000 high quality (HQ) reads. The assembly of the reads resulted in 19,410 contigs and 92,823 singletons. A large number of the differentially expressed genes identified were linked to ripening dependent processes including ethylene biosynthesis, perception and signalling, cell wall degradation and production of aromatic volatiles. In the banana fruit transcriptomes, we found transcripts included in 120 pathways described in the KEGG database for rice. The members of the expansin and xyloglucan transglycosylase/hydrolase (XTH) gene families were highly up-regulated during ripening, which suggests that they might play important roles in the softening of the fruit. Several genes involved in the synthesis of aromatic volatiles and members of transcription factor families previously reported to be involved in ripening were also identified.

**Conclusions:**

A large number of differentially regulated genes were identified during banana fruit ripening. Many of these are associated with cell wall degradation and synthesis of aromatic volatiles. A large number of differentially expressed genes did not align with any of the databases and might be novel genes in banana. These genes can be good candidates for future studies to establish their role in banana fruit ripening. The datasets developed in this study will help in developing strategies to manipulate banana fruit ripening and reduce post harvest losses.

**Electronic supplementary material:**

The online version of this article (doi:10.1186/s12870-014-0316-1) contains supplementary material, which is available to authorized users.

## Background

Banana fruit is the staple food for an estimated 400 million people. The banana plant is a large herbaceous, evergreen, flowering monocot belonging to the genus Musa (family Musaceae order Zingiberales). The majority of the cultivated banana is derived from the cross between *Musa acuminata* and *Musa balbisiana*. The fruit development and ripening is a complex process influenced by numerous factors including light, hormones, temperature and genotype. Ripening associated events in climacteric fruits, including banana, leads to developmentally and physiologically regulated changes in gene expression which ultimately bring changes in color, texture, flavor, and aroma of fruit [1-3]. Fruit ripening and softening involves irreversible physiological and biochemical changes which contribute to the perishability of the banana fruit. Premature ripening brings significant losses to both farmers and consumers alike. Therefore, there is an urgent need to develop tools to delay ripening and softening process through genetic engineering approaches.

Recently, the genome of banana was sequenced using DH-Pahang a double haploid (523 Mb) derived from a seedy diploid of the subspecies *M. malaccensis*, which led to the identification of 36,542 protein coding genes [4]. To support and accelerate genetic and genomic studies of banana, the banana genome hub was recently developed [5]. It has been commonly observed that ripening of banana involves extensive changes in the cell wall [6]. Earlier studies with banana identified multiple families of genes associated with cell wall degradation [7-11]. Apart from softening associated genes, a few genes have been identified in banana that relate to ethylene biosynthesis, signal transduction and transcription factors [12,13]. Approaches like subtractive hybridization and differential library screening have been employed [11,14-16] to identify differentially expressed genes during banana fruit ripening. However, apart from these genes, ripening likely involves the up and down-regulation of hundreds of genes not yet identified in banana.

Expressed Sequence Tags (ESTs) can be a useful tool for the purposes of gene discovery especially in non-model plants for which limited genomic information is available [17,18]. The in-depth generation of EST datasets and comparison provide information about all the expressed regions of a genome and can be used to characterize patterns of gene expression during fruit ripening. Using Next-Generation Sequencing (NGS) such databases have been developed and used for discovery and prediction of genes involved in fruit development and ripening. Transcriptome analyses in *Curcumas' melo* [19,20], citrus [21,22] blueberry [23], capsicum [24], Chinese bayberry [25], sweet orange [26], kiwi fruit [27], grape [28,29] tomato [30], watermelon [31] and many others have provided insight into genes and pathways involved in fruit development and ripening [32]. These databases are also a rich source of gene-derived molecular markers (e.g. simple sequence repeat, SSR) which can be used for germplasm breeding or physical mapping.

The primary objective of our study was to add to a basic understanding of banana fruit ripening at molecular level. In this study, we established a transcriptome datasets of unripe and ripe banana fruit using NGS technology based on 454 GS FLX Titanium platform. We identified genes involved in ethylene biosynthesis and its perception, fruit softening and other processes that initiate the ripening process to produce an edible banana fruit. The analysis has provided new information about many genes not previously identified that are expressed during banana fruit ripening. Some of these genes may be potential candidates that can be manipulated to increase the postharvest shelf life of banana and reduce economic losses. As a part of this study, we identified molecular markers for EST-SSRs that will facilitate marker-assisted breeding of banana. In addition, we mapped our reads to the *Musa acuminate* banana genome, as well as *de novo* assembly to account for the varietal difference in the species sequences. The contigs obtained were then mapped again to the banana genome to identify members of different gene families.

## Results and discussion

### Sequencing, annotation and mapping to the banana genome

To examine global changes occurring during ripening in the banana fruit, cDNA libraries from unripe and ripe banana fruit pulp (cultivar Harichhal) were sequenced using half plate run for each on a 454-GS FLX Titanium platform. Each transcriptome produced more than 7,00,000 high quality (HQ) reads (Table 1), which were assembled using the GS Assembler program as described in Material and methods.

**Table 1:** **Summary of** ***Musa acuminata*** **transcriptome sequencing, assembly and mapping**

**Sequencing details**	**Unripe**	**Ripe**
HQ Reads (bases)	763119 (197435772 bp)	720456 (186149403 bp)
Average length of Reads	258 bp	258 bp
**Combined assembly details**		
Total number of supercontigs	19410	
Total number of singletons	92823	
Number of bases	12460249	
Average contig size	642 bp	
N50	974	
**Mapping details**		
Total supercontigs mapped on CDS	15978	
Novel transcripts	3186	
**Annotation details**	**Contigs + Singletons**	
TAIR 9 pep	43337	
NR	23560	
TIGR	45022	
CDD	17959	

To study the differential expression of genes during banana fruit ripening, the total number of reads of unripe and ripe fruit transcriptomes were tagged, pooled and assembled using parameters described in material and methods using the GSAssembler program. A total of 14,83,544 reads were assembled into 19,410 contigs and 92,823 singletons. Within this assembly, 10,715 contigs were considered as large contigs with average size of 914 bp. The average contig length of all contigs was 642 bp with contig depth of 80 reads. These contigs and singletons were pooled together and are referred to here as the comparative transcripts. The total number of comparative transcripts was 1,12,233. As many gene families have multiple members, partially assembled transcipts could lead to erroneous results for differential analysis. To rule out this possibility, the combined assembly of unripe and ripe transcriptomes was preferred over the individually assembled transcripts of ripe and unripe transcriptomes. To annotate the comparative transcripts, transcripts were queried against the NCBI NR database, TAIR proteins, MSU Rice proteins using the BlastX program and against CDD using the rpsblast programme. The information about total number of comparative transcripts annotated by the different databases is provided in the Additional file 1, Additional file 2, Additional file 3, Additional file 4.

The assembled contigs were also mapped to the Musa genome to annotate the genes and also to study the differential expression in the two libraries. The 19,410 contigs and 92,823 singletons obtained were mapped to the 36,542 genes currently identified in the Musa genome. Of the total contigs and singletons, 15,978 contigs and 59,410 singletons mapped to 21,298 genes in the musa genome, and 8,490 of the mapped genes were common to both contigs and singletons. The remaining 3,432 contigs that did not match the Musa genome were annotated using the NCBI NR database, TAIR proteins, MSU7 version Rice proteins using the BlastX program and against CDD using the blastx programme. Of these, 247 contigs were annotated and the remaining 3,185 contigs were unique to the banana transcriptome. The 3,432 contigs which did not match the Musa genome may be due to differences between the genomic sequence of DH-Pahang and Harichhal varieties or transposable elements, experiment artefacts, or mis-prediction of genes in DH-Pahang. In addition, possibilities of post-transcriptional events like alternative splicing of the transcripts during ripening process leading to unique transcripts cannot be ruled out. Such alternative splicing during plant growth and development have been reported in other plants [33,34]. The 15,978 contigs matched to 12,315 Musa genes. Of these, 9,809 contigs had one CDS match in the Musa genome; whereas 6,169 contigs matched to 2,506 Musa CDS indicating that more than one contig mapped to the CDS sequences. This could be due to the partial contigs or due to alternative splicing of the transcript. To identify the alternative spliced transcripts, these 6,169 contigs and 2,506 Musa CDS were analysed as described in Material and Methods to identify alternatively spliced transcripts. It was found that 1,243 contigs that mapped to 402 CDS were alternatively spliced transcripts and 4,926 contigs that mapped to 2,104 Musa cds were partial transcripts.

### Comparative transcriptome analysis and differential gene expression

The number of reads in a particular contig is in general a measure of the transcript abundance of that particular contig, however this could also be due to sampling errors rather than genuine gene expression differences. To rule out this possibility, we applied three statistical tests P-value, FDR and the R statistical test. In the R statistical test [35] only R value > =8 was filtered that gave a believability of >99%. In this test, the singletons were statistically insignificant and hence discarded since the contigs were assembled from reads of unripe and ripe libraries. Using this statistic from 19,410 contigs, only 1,921 contigs were significantly differentially regulated. Of these, 653 genes were up-regulated (more than 2-fold) and 837 were down-regulated (more than 2-fold) in ripe fruit in comparison to unripe fruit (Additional file 5). Of these, 107 up-regulated and 83 down-regulated genes did not give hits in any of the databases analysed and could be novel genes that may be involved in different pathways or molecular networks during ripening in banana fruit. When analysis was carried out using differentially expressing genes during ripening in DH Pahang cultivar by D'Hont et al. [4], 353 genes showed differential expression. A large number of genes (98%) had similar expression pattern between our analysis and by D'Hont et al. (2012) [4]. A set of 569 differentially expressed genes had CDS counterpart in the Musa genome but were not significantly expressed in the earlier study [4]. These 569 differentially expressed genes may be playing an important role in the ripening of the banana variety Harichhal. To further annotate genes and study metabolic pathways and functional annotation, the KEGG description of TIGR and TAIR gene ids were transferred to the orthologous banana transcripts in our study.

### Genes involved in banana ripening

During banana fruit ripening, the pulp tissue losses its turgidity, softens and produces aromatic volitiles. To bring about these changes, a repertoire of genes is differentially expressed to regulate these processes. In the following sections, we have summarized changes in gene expression based on their predicted role in softening and aroma and flavor.

### Up-regulated genes during banana fruit ripening

#### Softening of the banana tissue

**Figure 1:**
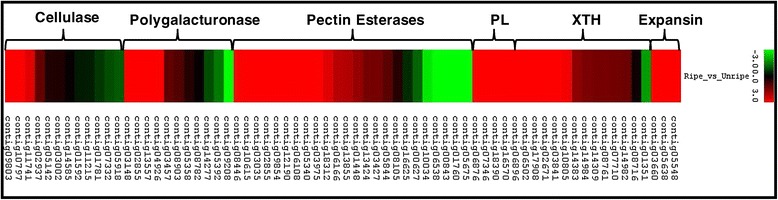
**Members of cell wall hydrolase gene families and change in expression in ripe and unripe fruit.** The color scale (representing log fold change values) is shown.

Cell wall hydrolysis plays an important role in plant growth and development that includes ripening as well as stress responses. Most of the genes involved in cell wall hydrolysis are members of multigene families and many have highly specialized functions in cell wall metabolism [36]. The process of softening begins with the onset of ripening. The stage at which the ripe tissue was collected for this study was fruit that had already begun to soften. It has been previously reported that the gene families responsible for softening of banana include expansins, pectate lyases and xylogulcan endotransglycosylases [6-9]. In the present study, several members of these gene families showed significantly higher expression in the ripe fruit compared to unripe fruit with some members of each family exhibiting more than a 12 fold increase in expression (Table 2). In our study, we analysed the expression of genes annotated as cellulase, polygalacturonase (PG), pectin esterase, pectate lyase (PL), XTH and expansin (Figure 1). We observed that the greatest increase in gene expression was associated with the gene families PL, XTH and expansin.

**Table 2:** **Top 50 up-regulated genes during fruit ripening process**

**Contigs**	**Fold change**	**Musa_ID**	**Description**
**contig08558**	**9.78**	**GSMUA_Achr5P07470_001**	**Expansin-A2**
**contig05638**	**9.7**	**GSMUA_Achr5P07470_001**	**Expansin-A2**
**contig03660**	**12.29**	**GSMUA_Achr11P22960_001**	**Expansin-A8**
**contig00739**	**8.09**	**GSMUA_Achr1P20310_001**	**Polygalacturonase QRT3**
contig19315	12.3	GSMUA_AchrUn_randomP04250_001	Probable pectate lyase 15
contig16570	8.22	GSMUA_AchrUn_randomP04250_001	Probable pectate lyase 15
contig06876	10.91	GSMUA_Achr6P28260_001	Probable pectate lyase 22
contig07346	9.88	GSMUA_Achr6P28260_001	Probable pectate lyase 22
contig18390	8.87	GSMUA_Achr6P28260_001	Probable pectate lyase 22
contig12687	9.67	GSMUA_Achr3P28030_001	NBS-LRR disease resistance protein, putative, expressed
contig08749	8.78	GSMUA_Achr3P15660_001	Putative Pathogenesis-related protein 1
**contig06502**	**11.13**	**GSMUA_AchrUn_randomP06130_001**	**Probable xyloglucan endotransglucosylase/hydrolase protein 32**
**contig17908**	**9.87**	**GSMUA_AchrUn_randomP06130_001**	**Probable xyloglucan endotransglucosylase/hydrolase protein 32**
contig00854	9.57	GSMUA_Achr5P14190_001	expressed protein
contig02218	8.46	GSMUA_Achr9P25300_001	expressed protein
contig00248	10.05	GSMUA_Achr2P03950_001	Formate dehydrogenase, mitochondrial
contig17026	9.74	GSMUA_Achr9P30640_001	Germin-like protein 12-1
contig00301	8.88	GSMUA_Achr11P06230_001	Glucan endo-1,3-beta-glucosidase 6
contig14270	8.02	GSMUA_Achr11P06790_001	Hydrolase, hydrolyzing O-glycosyl compounds, putative
contig17603	8.25	GSMUA_Achr5P28160_001	Hypothetical protein
contig06303	8.15	GSMUA_Achr2P08720_001	Non-symbiotic hemoglobin 2
contig01929	8.15	GSMUA_Achr2P05370_001	Nucleobase-ascorbate transporter 6
contig00487	9.95	GSMUA_Achr7P05830_001	Phototropin-1A
contig14617	9.23	GSMUA_Achr9P02950_001	Pleiotropic drug resistance protein 3
contig02025	9.65	GSMUA_Achr6P24140_001	Probable purple acid phosphatase 20
contig16011	9.16	GSMUA_Achr6P17340_001	Probable purple acid phosphatase 20
contig07941	10.09	GSMUA_Achr1P25050_001	Putative 3'-N-debenzoyl-2'-deoxytaxol N-benzoyltransferase
contig06446	8.37	GSMUA_Achr1P25050_001	Putative 3'-N-debenzoyl-2'-deoxytaxol N-benzoyltransferase
contig19360	10.34	GSMUA_Achr3P11750_001	Putative 3-oxoacyl-[acyl-carrier-protein] reductase
contig16157	10.12	GSMUA_Achr3P11750_001	Putative 3-oxoacyl-[acyl-carrier-protein] reductase
contig19172	9.94	GSMUA_Achr3P11750_001	Putative 3-oxoacyl-[acyl-carrier-protein] reductase
contig14749	9.68	GSMUA_Achr3P11750_001	Putative 3-oxoacyl-[acyl-carrier-protein] reductase
contig14752	9.65	GSMUA_Achr3P11750_001	Putative 3-oxoacyl-[acyl-carrier-protein] reductase
contig16111	9.02	GSMUA_Achr3P11750_001	Putative 3-oxoacyl-[acyl-carrier-protein] reductase
contig04351	8.79	GSMUA_Achr7P15630_001	Putative Avr9/Cf-9 rapidly elicited protein 132
contig10721	7.92	GSMUA_Achr5P03490_001	Putative Dihydroflavonol-4-reductase
contig13393	8.16	GSMUA_Achr9P00610_001	Putative expressed protein
contig17350	9.64	GSMUA_Achr4P16570_001	Putative O-methyltransferase ZRP4
contig17111	9.43	GSMUA_Achr4P16570_001	Putative O-methyltransferase ZRP4
contig17353	8.96	GSMUA_Achr3P11740_001	Putative Predicted protein
contig14200	8.57	GSMUA_Achr3P11740_001	Putative Predicted protein
contig00874	8.97	GSMUA_Achr5P28140_001	Putative Probable gibberellin receptor GID1L2
contig08936	8.01	GSMUA_Achr8P30810_001	Putative Probable receptor protein kinase TMK1
contig17237	10.21	GSMUA_Achr5P28140_001	Pyruvate decarboxylase isozyme 2
contig00472	8.1	GSMUA_Achr9P02950_001	Serine carboxypeptidase 3
contig00892	9.36	GSMUA_Achr2P20550_001	Zinc transporter 2
contig07019	10.22	GSMUA_Achr4P26810_001	14 kDa proline-rich protein DC2.15
contig04469	8.82	GSMUA_AchrUn_randomP23970_001	Cytochrome P450-1
contig06853	8.72	GSMUA_Achr6P03560_001	Putative Cytochrome P450 71B35
contig12549	9.11	GSMUA_Achr3P09170_001	Early nodulin-93

Five different expansin genes were identified in this study, and four of these were significantly up-regulated in the ripening fruit. From the XTH gene family, 13 members were identified of which several were significantly up-regulated in the ripening fruit. Since xyloglucan forms a major component of the cell wall in non-graminecious monocot plants, its role during ripening in banana is quite understandable. Members of XTH gene family have also been demonstrated to play important role in the ripening of other fleshy fruits like tomato and peach [37]. Similarly, 5 members were identified for the PL gene family and all of these were highly expressed during ripening.

Polygalacturonases and cellulases are also present as multigene families in banana. Some members of these families showed significantly up-regulation during ripening; however, it was generally not as high as members of the expansin, XTH and PL gene families. A few members of the PME gene family were also up-regulated; however, since one of the functions for PME is to modify pectins to make them more accessible to PL and PG, the transcripts for PME may have already declined in the ripe fruit (4-days post ethylene) used in the study. It has been reported that the highest PME activity is observed at 2 days post ethylene exposure and declined significantly by day 3 [6]. Details on the fold change of each gene family are provided in Additional file 6.

The beta glucosidases (GH family 17) are also known to play an important role in the softening of the banana fruit. As many as 7 beta glucosidases genes showed more than two fold enhanced expression in the ripe banana fruit as compared to unripe fruit in our analysis. Apart from its role in the cell wall degradation, beta glucosidases are also known to participate in the hydrolysis of phytohormones (i.e. glucosides of gibberellins, abscisic acid and cytokinins) and in the metabolism of cyanogenic glucosides. In graminae, these glucosides have been shown to be involved in the shikimate as well as aromatic acid biosynthesis pathways [38]. Genes related to the cell wall softening were among the top up-regulated genes indicating that softening of fruit as a major process during banana fruit ripening at molecular level.

#### Genes related to aroma and flavor compounds

**Figure 2:**
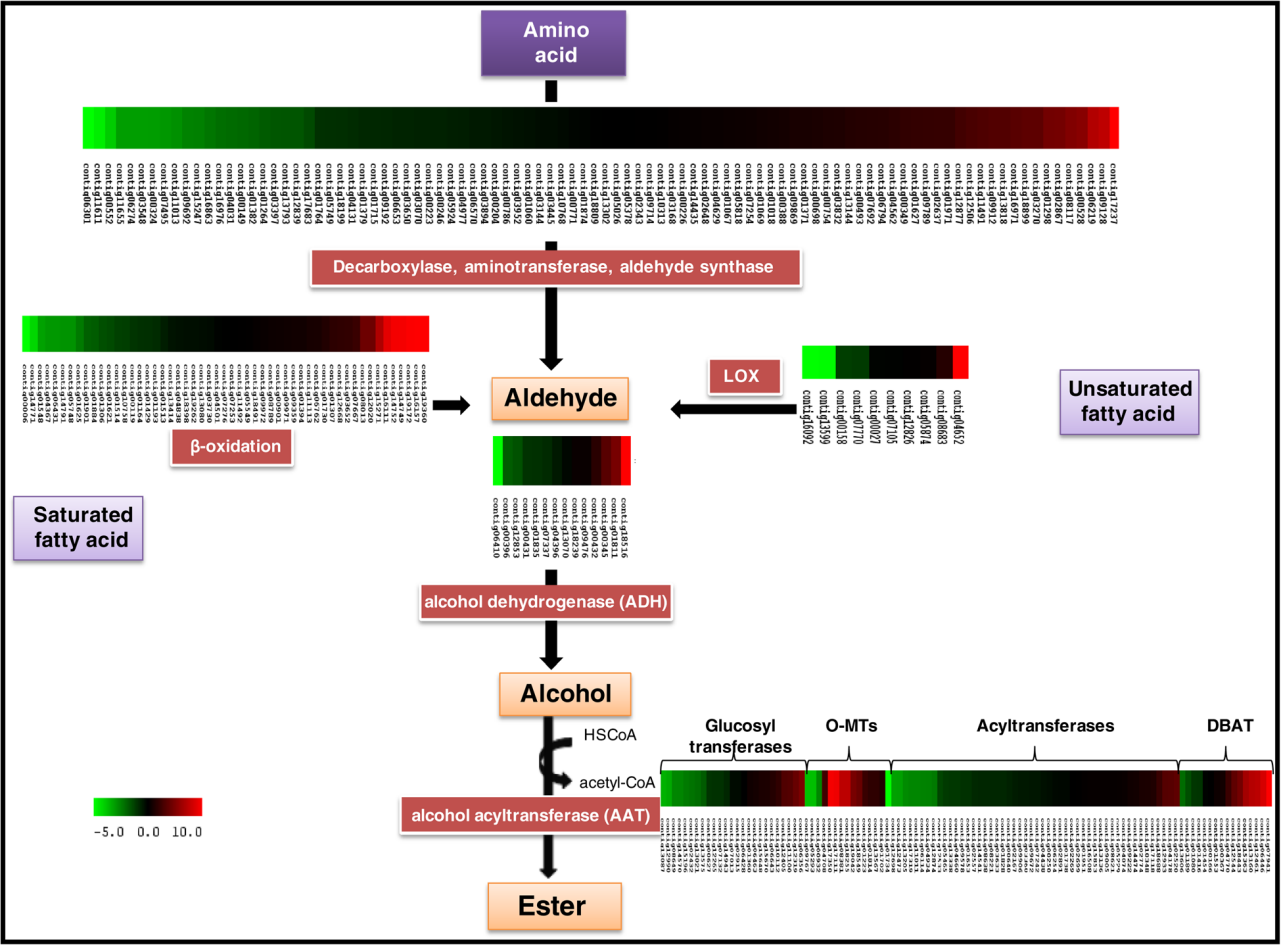
**Putative pathway and members of gene families involved in the synthesis of aromatic volatiles in banana during fruit ripening.** The color scale (representing log fold change values) is shown. LOX (lipoxygenases), HPL (Hydroperoxide lipase), DBAT (10-deacetylbaccatin III 10-O-acetyltransferase), 1-AGPATA (1-acyl-sn-glycerol-3-phosphate acyltransferase 1), DBTNBT (3-N-debenzoyl-2-deoxytaxol N-benzoyltransferase), COMT (chavicol O-methyltransferase), UFGT(flavonol-3-O-glycoside-7-O-glucosyltransferase 1), TAT ( taxadien-5-alpha-ol O-acetyltransferase).

The aroma of the banana fruit is attributed to the presence of various volatiles like isoamyl alcohol, isoamyl acetate, butyl acetate, elemecine and several others [39]. These volatiles are produced primarily by the phenylpropanoid pathway, fatty acid biosynthesis pathway and isoleucine biosynthesis pathway [40]. Since the major components of the aroma and flavor volatiles are esters, the expression of genes involved in biosynthesis of esters from amino acids, fatty acids and unsaturated fatty acids were analysed here. The genes involved in each step were identified (Figure 2) and differential expression was examined. The conversion of sugars to alcohol is mediated by ADH which is further converted to esters by AATs. At least 10 contigs annotated as ADH genes showed more than 2-fold up-regulation in the ripe fruit as compared to unripe fruit. Similarly, the lipoxygenases genes were also significantly up-regulated in the ripe fruit as compared to unripe fruit. A large number of transferases were up-regulated in the ripe sample, which could be playing a putative role in the production of the aroma volatiles.

Our analysis also suggested that genes for the butyltransferases, acetyltransferases, O-methyltransferases were significantly up-regulated in the ripe fruit as compared to unripe fruit (Table 3). The members of BAHD acyltransferases gene family are known to be involved in the acetyl CoA dependent acylation of secondary metabolites resulting in the formation of esters and amides. Hoffmann et al., [41] categorised these in four different groups namely (A) Taxus acyltransferase involved in taxol biosynthesis (B) anthocyanin acyltransferases involved in anthocyanin biosynthesis (C) enzymes with un-related substrates and (D) hydroxycinnamoyl acyltransferase. In the present study, at least 30 acyltransferases were significantly up-regulated in the ripe fruit. One of the gene annotated as 3-N-debenzoyl-2-deoxytaxol N-benzoyltransferase was one of the most highly up-regulated genes (10-fold) in the ripe fruit. This enzyme family is involved in the acylation of the final step in the taxol biosynthesis pathway. The hydroxycinnamoyl acyltransferase also showed a significant increase (5.8-fold) in the ripe fruit (Additional file 6). The significatly higher expression of these genes in the ripe fruit suggests their involvement in the production of banana volatile esters that may contribute to the ripe fruit aroma. The role of AAT has already been established in the ester formation [42]. A set of other genes including 4-coumarate--CoA ligase 1, peroxisomal-coenzyme A synthetase involved in the formation of aromatic volatiles were also up-regulated in ripe fruit (Table 2 and Additional file 6). Our analysis indicates that volatile esters are generally synthesized from amino acids and not the fatty acid degradation pathway (Figure 2).

**Table 3:** **Top 50 down-regulated genes during fruit ripening process**

**Contigs**	**Fold change**	**Musa_ID**	**Description**
contig00798	6.48	GSMUA_Achr5T15680_001	Putative Cytochrome P450 86B1
contig02008	8.81	GSMUA_Achr6T33140_001	Putative Ethylene-responsive transcription factor RAP2-7
contig02568	7.81	GSMUA_Achr6T33140_001	Putative Ethylene-responsive transcription factor RAP2-7
contig08797	9.49	GSMUA_Achr6T27190_001	Glucose-1-phosphate adenylyltransferase large subunit 2,
contig16906	10.11	GSMUA_Achr4T33530_001	Glucose-6-phosphate/phosphate translocator 2, chloroplast
contig04246	9.33	GSMUA_Achr4T33530_001	Glucose-6-phosphate/phosphate translocator 2, chloroplast
contig03057	6.4	GSMUA_Achr8T07300_001	Glucose-6-phosphate/phosphate translocator 2, chloroplast
contig00800	8.89	GSMUA_Achr10T29580_001	40S ribosomal protein S3-3
contig01027	6.39	GSMUA_Achr6T31150_001	60S ribosomal protein L15
contig04831	6.62	GSMUA_Achr3T31330_001	60S ribosomal protein L18a-2
contig16188	6.8	GSMUA_Achr2T16990_001	ADP,ATP carrier protein 1, chloroplastic
contig00923	6.97	GSMUA_Achr5T07760_001	ADP-ribosylation factor 2
contig00295	7.2	GSMUA_Achr9T15680_001	Alpha-glucan water dikinase 2
contig02907	8.54	GSMUA_Achr9T06260_001	Aquaporin TIP4-4
contig01324	6.34	GSMUA_Achr10T18110_001	Aspartate-semialdehyde dehydrogenase
contig01110	6.38	GSMUA_Achr10T00360_001	Calmodulin
contig00548	7.37	GSMUA_Achr9T06150_001	CCT motif family protein, expressed
contig01960	6.82	GSMUA_Achr9T06150_001	CCT motif family protein, expressed
contig10082	8.11	GSMUA_Achr1T01000_001	expressed protein
contig05110	6.46	GSMUA_Achr2T15930_001	expressed protein
contig16640	9.95	GSMUA_Achr10T01990_001	Hypothetical protein
contig00120	7.66	GSMUA_Achr7T00770_001	Hypothetical protein
contig06596	7.49	GSMUA_Achr2T14210_001	Hypothetical protein
contig07709	7.32	GSMUA_AchrUn_randomT28490_001	Hypothetical protein
contig04324	6.46	GSMUA_Achr1T01050_001	integral membrane transporter family protein
contig16958	6.46	GSMUA_Achr1T02850_001	Monosaccharide-sensing protein 2
contig03813	6.34	GSMUA_Achr1T02850_001	Monosaccharide-sensing protein 2
contig02994	6.32	GSMUA_Achr7T21780_001	NAC domain-containing protein 68
contig03243	6.6	GSMUA_Achr8T12920_001	Probable aquaporin TIP1-1
contig00764	7.77	GSMUA_Achr3T24740_001	Putative Cathepsin B
contig03213	6.37	GSMUA_Achr3T06220_001	Putative expressed protein
contig01856	6.39	GSMUA_Achr4T16020_001	Putative Levodione reductase
contig00940	8.53	GSMUA_AchrUn_randomT26730_001	Putative Pathogenesis-related protein 1
contig00222	7.1	GSMUA_Achr11T00570_001	Putative Protein disulfide-isomerase
contig00491	8.59	GSMUA_Achr2T20210_001	Putative Pyruvate kinase, cytosolic isozyme
contig01098	6.39	GSMUA_Achr7T14740_001	Putative Receptor-like protein kinase HSL1
contig07118	7.54	GSMUA_Achr9P20500_001	Putative uncharacterized protein
contig08848	8.02	GSMUA_Achr9P22830_001	Putative Zinc finger protein 2
contig17826	10.41	GSMUA_Achr6T02890_001	Pyrophosphate-energized vacuolar membrane proton pump
contig17777	10	GSMUA_Achr7T20850_001	Pyrophosphate-energized vacuolar membrane proton pump
contig10985	7.24	GSMUA_Achr7T20850_001	Pyrophosphate-energized vacuolar membrane proton pump
contig02678	6.71	GSMUA_Achr8T34150_001	Rhodanese-like domain containing protein, putative
contig00812	6.68	GSMUA_Achr3T11670_001	RNA polymerase I specific transcription initiation facto
contig11125	6.52	GSMUA_AchrUn_randomT07990_001	SNF1-related protein kinase regulatory subunit beta-1
contig04585	6.43	GSMUA_Achr11T04500_001	S-norcoclaurine synthase 1
contig00394	6.77	GSMUA_Achr2T12390_001	Tubulin alpha-3 chain
contig01434	7.76	GSMUA_Achr9T30160_001	Ubiquitin-60S ribosomal protein L40
contig01831	7.88	GSMUA_Achr1T28140_001	Vacuolar-processing enzyme
contig00321	6.39	GSMUA_Achr4T28430_001	YT521-B-like family domain containing protein, expressed
contig08692	6.37	GSMUA_Achr4T24460_001	ZOS2-16 - C2H2 zinc finger protein

### Down-regulated genes during banana fruit ripening

As the fruit matures for ripening, the genes which are required for the growth and development are not required and are therefore down-regulated. We carried out analysis to identify such genes using comparative transcriptome data. The vacuolar ATP transporters play an important role during the development of fruit and are known to be helpful in creating a proton gradient across the tonoplast membrane, which is effective in transport of nutrients, metabolites and proteins. As the process of softening starts, these proteins are no longer required and hence the gene encoding V-ATPases, showed a significant decline in their expression in ripe fruit as compared to unripe fruit. In the present study, the most significantly down-regulated genes were the trans-membrane transporters and antiporters. Out of these expression of AVP1, a gene encoding an ATPase/hydrogen-translocating pyrophosphatase, decreased in ripe fruit compared to unripe fruit by 12-fold, the greatest decline of any transcript in our analysis (Table 3). These genes are mainly involved in maintaining the pH balance and transport of important metabolites. As ripening proceeds, the fruit vacuolar membrane starts to degenerate as these types of transporters may not be required. As many as 112 genes annotated as transporters in various families were down-regulated (Additional file 5).

In our analysis, many of the genes responsible for RNA processing and protein synthesis were down-regulated in ripe fruit. In addtion, a large number of transcription factors and genes associated with flower and fruit development were down-regulated. We observed a decline in expression of the several floral homeotic genes, FT genes, auxin responsive genes in ripe fruit. These regulatory proteins may no longer be required at ripening stage hence, showed a significant reduction in gene expression in ripe fruit as compared to unripe fruit.

### Modulated pathways during banana fruit ripening

The KO ids of all the contigs that matched with TAIR ids were extracted and involvement of genes in different pathways was analysed using KEGG pathway database. Analysis suggested that the transcriptomes of both the unripe and ripe fruit pulp included genes associated with many different KEGG pathways. The genes from banana were mapped onto the KEGG pathway under metabolism, genetic information processing, environmental information processing, cellular processes and organisms systems. Metabolic pathways identified included carbohydrate, lipid, amino-acid, nucleotide, energy metabolisms. The KEGG pathways database for the rice genome has 120 pathways and genes for each of these pathways were identified in banana (Additional file 7), indicating the complete coverage of the transcriptomes in our study. GO analysis of differentially expressed genes indicated that most of the ripening asscociated gene expression was assigned to funtional groups for transcription factors, nucleic acid activity and receptor binding activity. More than 50 percent the transcripts in the transcriptomes were involved in energy pathways, hydrolase activity, response to abiotic and biotic stimulus and other biological processes. These are some of the pathways that were active during ripening and this data might provide a platform to explore ripening related genes (Additional file 8).

**Figure 3:**
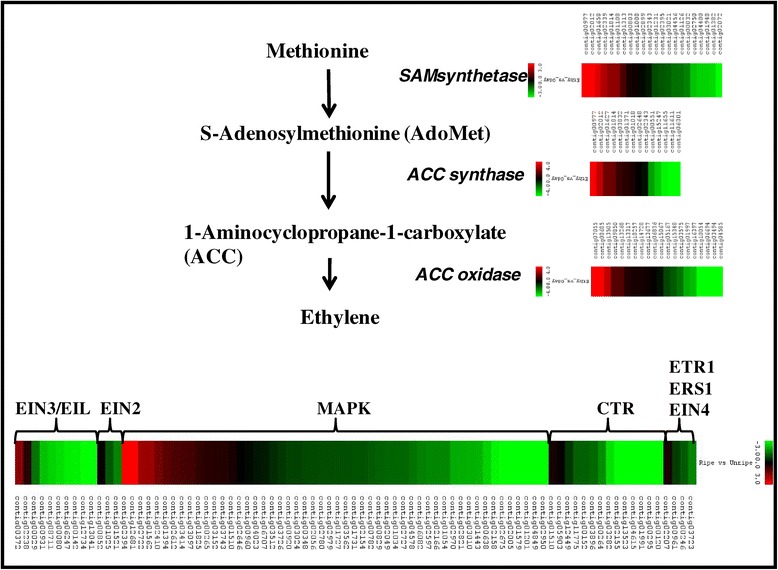
**Selected members of gene families involved in ethylene biosynthesis and perception and their differential expression during banana fruit ripening.** The color scale (representing log fold change values) is shown at each step.

As ethylene biosynthesis and perception is essential to banana fruit ripening, a comprehensive analysis for the genes involved in ethylene synthesis and signal transduciton was carried out. Several contigs were identified as gene related to ethylene biosynthesis including SAM, ACS and ACO (Figure 3). Various members of the each gene family showed differential gene expression in ripe and unripe fruit. As each of these gene families has several members, expression of some genes was up-regulated while others was either down-regulated or remained unchanged. It might be assumed that the genes that were up-regulated were associated with system 2 ethylene biosynthesis whereas those that were down-regulated were linked to system 1 ethylene biosynthesis or other biological processes [43]. In addition, a large number of genes associated to the ethylene signal transduction were also identified in our analysis. Many of these genes have been identified for the first time in banana as well. As many as 14 members related to CTR1 and CTR1-like are identified in our study. Similarly, genes related to ETR1, ERS, EIN2, EIN3, EIN4, EIL were also identified in the transcriptome database. In another study, through genome-wide analysis, 25 members of MAPK were also identified. Of these, many were differentially regulated [44] and could hold the key to finding the missing members of the ethylene signal transduction pathway during fruit ripening.

### Transcription factors and their role in ripening

Gene regulation through transcription factors (TFs) plays an important role in biological and cellular processes. To study a potential role for the transcription factors in banana fruit ripening, all the genes in the plant transcription factor (TF) database [45] were downloaded and queried against the supercontigs in banana transcriptome using the blastx program. The plant TF database has 29,473 sequences classified in 74 TF gene families. Using a lower limit for an acceptable e-value of 10^−10^, we identified 74 different TF gene families represented in our combined transcriptome (Table 4). The most abundant TFs were related to the C3H, MADS, MYB-related, bZIP, NAC, WRKY gene families. These TFs are encoded by multigene families in plants and it is likely that these are present as multigene family in banana. Some of the MADS, bHLH, WRKY, AP2-EREBP, MYB-related and NAC domain TF families were highly expressed in ripe fruit. The MADS domain transcription factors are reported to be involved in various processes of fruit ripening [3,12,43,46]. At the ripe fruit stage we collected, the most important processes are of cell wall degradation and synthesis of aromatic volatiles. The MADS and NAC domain proteins are known to interact with each other and other cell wall related gene promoters like expansin and others [43]. Since most of these TFs belong to multigene families, many TFs were down regulated during ripening, indicating their differential role during various stages of ripening and fruit development.

**Table 4:** **Transcription factor gene families and their members in banana fruit transcriptomes**

***TF family***	***Unripe***	***Ripe***	***TF family***	***Unripe***	***Ripe***	***TF family***	***Unripe***	***Ripe***	***TF family***	***Unripe***	***Ripe***
**ABI3VP1**	**34**	**31**	**CAMTA**	**18**	**16**	**LFY**	**0**	**0**	**SBP**	**43**	**54**
**Alfin-like**	**20**	**16**	**CCAAT**	**45**	**39**	**LIM**	**4**	**5**	**Sigma70-like**	**12**	**13**
**AP2-EREBP**	**98**	**89**	**CPP**	**5**	**0**	**LOB**	**14**	**15**	**SRS**	**2**	**1**
**ARF**	**76**	**29**	**CSD**	**5**	**8**	**MADS**	**191**	**166**	**TAZ**	**5**	**8**
**ARR-B**	**2**	**2**	**DBP**	**53**	**71**	**mTERF**	**80**	**77**	**TCP**	**10**	**17**
**BBR/BPC**	**10**	**7**	**E2F-DP**	**10**	**12**	**MYB**	**54**	**43**	**Tify**	**34**	**42**
**BES1**	**22**	**12**	**EIL**	**20**	**24**	**MYB-related**	**155**	**168**	**TIG**	**0**	**5**
**bHLH**	**172**	**192**	**FAR1**	**140**	**135**	**NAC**	**120**	**154**	**Trihelix**	**38**	**39**
**BSD**	**23**	**35**	**FHA**	**85**	**120**	**NOZZLE**	**0**	**0**	**TUB**	**35**	**26**
**bZIP**	**144**	**119**	**G2-like**	**58**	**63**	**OFP**	**5**	**9**	**ULT**	**0**	**1**
**C2C2-CO-like**	**16**	**6**	**GeBP**	**12**	**7**	**Orphans**	**119**	**133**	**VARL**	**0**	**0**
**C2C2-Dof**	**30**	**23**	**GRAS**	**79**	**63**	**PBF-2-like**	**4**	**9**	**VOZ**	**3**	**5**
**C2C2-GATA**	**27**	**39**	**GRF**	**5**	**3**	**PLATZ**	**6**	**4**	**WRKY**	**93**	**89**
**C2C2-YABBY**	**6**	**3**	**HB**	**170**	**128**	**RWP-RK**	**21**	**30**	**zf-HD**	**2**	**1**
**C2H2**	**133**	**157**	**HRT**	**5**	**3**	**S1Fa-like**	**0**	**1**	**Zn-clus**	**0**	**0**
**C3H**	**331**	**340**	**HSF**	**22**	**14**	**SAP**	**0**	**0**			
**Other Transcriptional regulators:**											
***TF family***	***Unripe***	***Ripe***	***TF family***	***Unripe***	***Ripe***	***TF family***	***Unripe***	***Ripe***	***TF family***	***Unripe***	***Ripe***
**ARID**	**20**	**13**	**IWS1**	**2**	**1**	**PHD**	**218**	**203**	**SOH1**	**2**	**0**
**AUX/IAA**	**67**	**73**	**Jumonji**	**25**	**17**	**Pseudo ARR-B**	**0**	**0**	**SWI/SNF-BAF60b**	**25**	**21**
**Coactivator p15**	**0**	**0**	**LUG**	**21**	**18**	**RB**	**2**	**3**	**SWI/SNF-SWI3**	**11**	**4**
**DDT**	**9**	**13**	**MBF1**	**4**	**2**	**Rcd1-like**	**4**	**6**	**TRAF**	**80**	**96**
**GNAT**	**80**	**88**	**MED6**	**0**	**4**	**SET**	**136**	**120**			

### Novel genes with modulated expression during banana fruit ripening

A large number of genes that did not show any hits to any of the databases but were significantly and differentially regulated were identified in this study (Additional file 9). These genes could be involved in the various processes like cell-wall softening, production of aromatic volatiles, changes in colour of the peel and development of flavour compounds. A total of 3185 genes did not show any hits to any of the databases (NR, AGIprot, Rice, CDD) of these 548 and 648 genes were 2-fold up- and down-regulated respectively.

### Validation of differential gene expression

**Figure 4:**
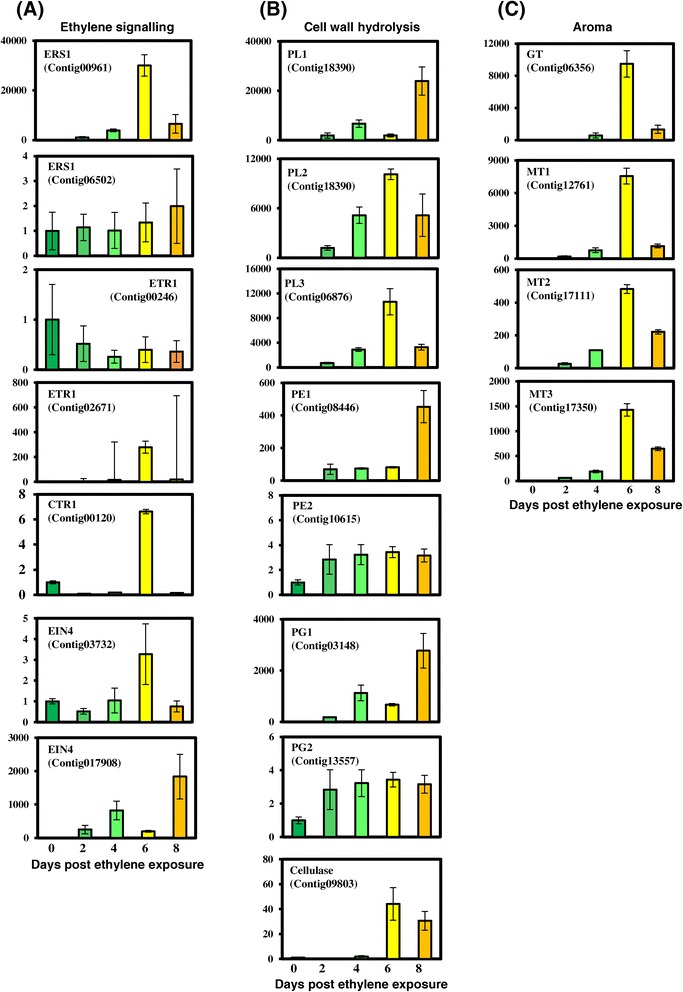
**The expression profiles for selected members of gene families associated with (A) Ethylene perception and signaling (B) cell wall modification and (C) aroma formation.** Quantitative real time PCR of the gene families was carried out using total RNA isolated from fruit tissues. 0 to 8 represent the days post ethylene treatment in the banana fruits. The relative transcript abundance was normalised using banana actin gene.

The differential expression of a few selected genes was confirmed by RT-qPCR. These genes were randomly selected from three categories including genes related to the ethylene signalling, aroma and softening. The expressions for each gene was examined in unripe fruit (0) and 2, 4, 6 and 8 days post ethylene treatment (Figure 4). In regard to genes related to ethylene signalling, of the ethylene receptor genes examined, expression of an ERS1-like gene and an EIN4-like gene increased markedly (>10-fold) during ripening. The CTR1 gene, which is downstream from the ethylene-receptors, initially showed a reduction in expression in the early stages of ripening, but had a significant increase in expression at 6 days post ethylene exposure (Figure 4). Similarly, the ETR1 gene showed a reduction in expression at day 2, which later increased at 6 days post ethylene exposure. Out of all the genes selected for analysis, one of the ERS1 genes did not show significant change in expression and the EIN4 gene showed a down-regulation during ripening process. The differential expression of these genes as analysed through quantitative real time PCR was similar to that observed in the comparative transcriptome analysis. The aroma related GTs and MTs showed a significant increase in expression as the ripening progressed, and this increase in expression generally began at day 4 and reached a maximum at day 6 of ripening. Expression of the aroma genes appears to correlated with the stage when the fruit emits a characteristic aroma and after this senescence and over-ripening sets in resulting in a less palitable fruit. The aroma volatiles are no longer needed and hence the expression of these genes starts to decrease.

For the softening related genes the expression of selected members of PE, PL XTH, Cellulase and PG gene families were studied. As observed in comparative transcriptome data, quantitative-RT analysis also suggested significantly higher expression of XTH and PL genes as the ripening progressed. The expression of these genes started increasing drastically at the 4 day stage and continued till senescence of the fruit. The expression of one member of cellulase and 2 members of PG gene families were also studied through quantitative-RT analysis. The expression of these genes increased during the progress of ripening, however, it was not as significant as the increase in the XTH, PL and PE genes. The results obtained through quantitative-RT analysis verified and extended differential expression as observed in the comparative transcriptome analysis between ripe and unripe fruit.

### SSR markers

EST derived SSR markers are an important tool for gene mapping. SSR marker studies have been done in banana earlier and a banana SSR database is available; however, identification of SSRs was done using the publicly available ESTs, which was somewhat limited for banana. To enrich the SSR markers in Banana, we identified SSRs using the Misa pipeline in the combined assembly data of the ripe and unripe transcriptomes (Table 5). The combined transcriptome was screened for the presence of di-, tri-, tetra-, penta- and hexa- nucleotide SSR motifs and 1,042 SSRs were identified in the Supercontigs for the unripe and ripe fruit transcriptomes. The Di- and tri- repeats formed the major part of SSRs and were around 70% of the total SSRs identified. The annotation of the contigs associated with different SSRs was extracted using a custom perl script. Several of the SSRs were in genes up-regulated in ripening process. Contig17908 and Contig03660, which containined one SSR each, were annotated as expansin and XTH, respectively, and both were strongly up-regulated during ripening (Additional file 10). The SSRs identified, in this study, will be useful as genetic markers for breeding improved varieties of banana.

**Table 5:** **SSRs identified in assembled contigs of** ***Musa acuminata***

**Description**	**Contigs**	**Singletons**
Total number of sequences examined	19410	92,823
Total size of examined sequences (bp)	12460249	13532481
Total number of identified SSRs	1106	1930
Number of SSR containing sequences	1004	1772
Number of sequences containing more than 1 SSR	94	141
Di-nucleotide repeats	454	834
Tri-nucleotide repeats	536	579
Tetra-nucleotide repeats	24	49
Penta-nucleotide repeats	5	5
Hexa-nucleotide repeats	8	8

## Conclusion

Banana is an economically important fruit in many parts of the world; however, huge post-harvest losses are incurred by farmers and consumers due to over-ripening. The ethylene regulated ripening in banana has not been studied in great detail at the molecular level. Most of the studies carried out are related to single genes or a single gene family. However, ten gene families related to ethylene biosynthesis and signalling have been studied recently in detail [47]. More global analysis of gene expression in banana has been restricted to subtractive hybridisation and PAGE-DDGE, both of which fail to give a comprehensive picture of the transcriptome. In the present study, we have sequenced the transcriptomes of two stages of the banana fruit pulp and identified genes involved in the ripening processes. The two most important processes related to banana fruit ripening were softening and production of aroma volatiles. Both of these processes were studied in detail and many genes related to aroma formation were identified. Several acyltransferases were identified that are likely involved in the synthesis aromatic volatiles and flavour components. In addition, the present study highlights the importance of expansins, PL and XTH in the softening of the fruit. Apart from enriching the banana genes in the database, we have also identified many novel genes that could be playing an integral part during ripening in banana, and may be good candidates for future gene manipulation studies.

## Methods

### Plant material and RNA isolation

Fruits of *Musa accuminata* (Dwarf Cavendish, Genome AAA, var. Robusta, Harichhal, germplasm code TRY0081 at National Research Centre for Banana, India) were harvested from plants grown in the field of CSIR-National Botanical Research Institute, Lucknow. Fruits were washed, wiped and exposed to 100 μL/L ethylene for 24 h to initiate ripening and stored for four days as described earlier [6]. The selection of fruit, ethylene treatment and RNA isolation was replicated four time using ten fruits in each experiment. Two fruits from each set were randomly chosen and the pulp pooled and frozen in liquid nitrogen and stored in −70°C for further use. Frozen tissues from ripe and unripe fruits were ground to a fine powder in liquid nitrogen using a mortar and pestle. Total RNA from unripe and ripe tissues was extracted using method previously described [48] followed by DNaseI treatment according to manufacturer’s instructions (Ambion, USA). RNA quality was checked on agarose/EtBr gel and quantity determined with a spectrophotometer (Nanodrop, Thermo Scientific, USA).

### cDNA Library construction and 454 sequencing

An equal amount of total RNA from each of the four different preparations was pooled and used for library preparations. First strand cDNA was prepared using 5 μg of the pooled RNA using oligo-dT primer and Superscript II reverse transcriptase (Invitrogen, Carlsbad, CA). A double-stranded cDNA library was then synthesized as described in double stranded cDNA synthesis kit (Invitrogen, Carlsbad, CA), and the double-stranded cDNA purified by Gene Chip Sample Cleanup Module (Affymetrix, USA). Quantity as well as quality of the double stranded cDNA library was checked on an Agilent 2100 Bioanalyzer DNA chip (Agilent Technologies Inc., Santa Clara, CA). Approximately three micrograms of double-stranded cDNA was sheared by nebulization to produce random fragments of about 250–800 bp in length. The nebulized cDNA was purified further using QIAGEN QIA quick PCR purification spin columns and pooled. Fragments smaller than 300 bp were removed and the purified cDNA samples were assesed on DNA chip (Agilent 2100 Bioanalyzer, USA) to analyze quantity as well as confirm the fragment size (350–800 bp). Adapter ligation and purification of adapter ligated library was done according to manufacturer’s instruction (Roche, USA). The quality and quantity of library was evaluated on Agilent High sensitivity chip and spectroflurometer (Perkin Elmer, USA), respectively. The double-stranded cDNA fragments were then denatured to generate single-stranded cDNA fragments, which were then amplified by emulsion PCR for sequencing according to manufacturer’s instructions (454 Life Sciences, Roche, USA). Reads from unripe and ripe libraries were processed and trimmed to remove low quality and primer sequences.

### De novo sequence assembly and annotation

The raw 454 sequences from ripe and unripe banana fruit libraries were screened and trimmed for weak signals by GS FLX pyrosequencing software to yield high-quality (HQ) sequences (>99.5% accuracy of single-base reads). The primer and adapter sequences were trimmed from the HQ sequences, and sequences shorter than 50 bp removed before assembly. The trimmed sequences were assembled into unique contigs and singletons using ROCHE GS Assembler (version 2.5.3) with 40 base pair overlap and 96% identity. The contigs and singletons were annotated using a standalone version of NCBI BLASTx program [49] against the Arabidopsis protein database at The Arabidopsis Information Resource (TAIR; http://www.arabidopsis.org) (version Tair9), MSU Rice genome annotation and the NCBI non-redundant protein (Nr) database (http://www.ncbi.nlm.nih.gov; released on 06/23/2009) and The Banana Genome Hub (http://banana-genome.cirad.fr/) using the BLASTx algorithm with an E-value cut-off of 10^−5^ and extracting only the top hit for each sequence. Annotation against the CDD database (http://www.ncbi.nlm.nih.gov) was done using the rpsblast programe of the blast suite, and pfam using the hmmer v 3 programe. To find out the potential coding regions in unigenes were presented or not, ESTScan was carried out using HMM based program. To analyse the partial and alternative transcripts, the contigs were computationally fragmented to 100 bp tagged and mapped to the banana genome using the bowtie2 programme [50]. Parts of the contigs that skipped an exon during mapping were identified as alternatively spliced mapping on banana genome [4].

### Functional classification and biological pathways assignment

To gain an understanding of metabolic and genetic networks operating during ripening, the genes identified in our transcriptome were mapped according to their linkage in the Kyoto Encyclopedia of Genes and Genomes (KEGG) pathways database. Enzyme commission (EC) numbers were assigned to unique sequences, based on the BLASTx search of protein databases, using a cut off E-value 10^−5^. The output of KEGG analysis includes KEGG orthology (KO) assignments and KEGG pathways (http://www.genome.jp/kegg/) that are populated with the KO assignments. Gene ontology (GO) analysis was also performed using the GO terms indentified for banana supercontigs having an E-value of >10^−5^ in a BLAST search of Arabidopsis genes in the TAIR databases.

### Digital gene expression and pathway analysis

To analyse differential gene expression the reads per contigs were counted and the transcript per million calculated. Differentially expressed genes were identified using DESeq package [51]. To statistically determine the differential gene expression the R statistics [35] was applied, and R ≥8 were considered to be highly significant. To calculate the threshold R value, 1000 datasets for each library was generated according to the random Poisson distribution as previously described [35]. For the comparative expression analysis with the musa genome, all the unigenes including singletons were mapped to annotated gene models predicted for the musa genome. Expression levels were calculated using TPM (Transcripts per million) of contigs and the predicted levels checked again using the DESeq pacakge [51]. Pathway analysis was performed using the KEGG and Biocyc program for Arabidopsis and Rice, and the contigs were fished using custom made perl scripts. Clustering of the genes and the heat maps were generated using the MEV software (http://www.tm4.org/mev.html).

### Designing of oligonucleotide primers and real-time PCR analysis

A set of oligonucleotide primers (Additional file 11) were designed for RT-qPCR on the basis of sequence information developed through sequence analysis. For RT-qPCR, first-strand cDNA was synthesized using total RNA in a Revert Aid H minus first strand cDNA synthesis kit (Fermentas life Sciences, USA) according to the prescribed protocol. The cDNA was checked by semi quantitative PCR, followed by agarose gel electrophoresis. The PCR mix for Real time PCR contained 1 μl of diluted cDNA (10 ng), 10 μl of 2× SYBR Green PCR Master Mix (Applied Biosystems, USA), and 200 nM of each gene-specific primer in a final volume of 20 μl. A no template control was also performed for each primer pair. Expression was quantified using the Applied Biosystems 7500 Fast Real time PCR System. All the PCRs were performed under following conditions: 20 sec at 95°C, 3 sec at 95°C, and 40 cycles of 30 sec at 60°C in 96-well optical reaction plates (Applied Biosystems, USA). The specificity of amplicons was verified by melting curve analysis (60°C to 95°C) after 40 cycles. Three technical replicates were performed for each cDNA.

### Availability of supporting data

The data sets supporting the results of this article are available in the NCBI GenBank repository [http://www.ncbi.nlm.nih.gov/bioproject/?term=PRJNA172246] and in the NCBI SRA repository [http://www.ncbi.nlm.nih.gov/sra/?term=SRA057081].

## Additional files

Additional file 1:
**Comparative transcripts queried against the CDD database at NCBI.**
Additional file 2:
**Comparative transcripts queried against the NCBI NR database.**
Additional file 3:
**Comparative transcripts queried against the TIGR Rice protein database.**
Additional file 4:
**Comparative transcripts queried against the Arabidopsis AGI protein database.**
Additional file 5:
**Calculation of fold change and R value to identify genes differentially expressing in the Unripe and Ripe transcriptome library.**
Additional file 6:
**Fold change calculations of genes for which the heat map has been made in the different figures.**
Additional file 7:
**List of the Rice pathways present in the KEGG database with the number of genes present in the** ***Musa acuminata*** **comparative transcripts.**
Additional file 8:
**GO annotation of differentially regulated genes in banana transcriptome.**
Additional file 9:
**List of novel genes identified in this study in the comparative transcriptome.**
Additional file 10:
**Description of genes associated with identified SSRs.**
Additional file 11:
**List of primers and their sequences used in RT-qPCR.**

## References

[1] Bapat VA, Trivedi PK, Ghosh A, Sane VA, Ganapathi TR, Nath P (2010). Ripening of fleshy fruit: molecular insight and the role of ethylene. Biotechnol Adv.

[2] Klee HJ, Giovannoni JJ (2011). Genetics and control of tomato fruit ripening and quality attributes. Annu Rev Genet.

[3] Nath P, Sane VA, Sane AP, Trivedi PK, RA M (2005). Regulation of Plant Gene Expression. Encylopedia of Molecular and Cell Biology and Molecular Medicine.

[4] D'Hont A, Denoeud F, Aury JM, Baurens FC, Carreel F, Garsmeur O, Noel B, Bocs S, Droc G, Rouard M, Da Silva C, Jabbari K, Cardi C, Poulain J, Souquet M, Labadie K, Jourda C, Lengellé J, Rodier-Goud M, Alberti A, Bernard M, Correa M, Ayyampalayam S, Mckain MR, Leebens-Mack J, Burgess D, Freeling M, Mbéguié-A-Mbéguié D, Chabannes M, Wicker T (2012). The banana (*Musa acuminata*) genome and the evolution of monocotyledonous plants. Nature.

[5] Droc G, Larivière D, Guignon V, Yahiaoui N, This D, Garsmeur O, Dereeper A, Hamelin C, Argout X, Dufayard JF, Lengelle J, Baurens FC, Cenci A, Pitollat B, D'Hont A, Ruiz M, Rouard M, Bocs S (2013). The banana genome hub. Database (oxford).

[6] Lohani S, Trivedi PK, Nath P (2004). Changes in activities of cell wall hydrolases during ethylene-induced ripening in banana: effect of 1-MCP, ABA and IAA. Postharvest Biol Technol.

[7] Asif MH, Nath P (2005). Expression of multiple forms of polygalacturonase gene during ripening in banana fruit. Plant Physiol Biochem.

[8] Trivedi PK, Nath P (2004). MaExp1, an ethylene-induced expansin from ripening banana fruit. Plant Sci.

[9] Asha, Sane VA, Sane AP, Nath P (2007). Multiple forms of Î±−expansin genes are expressed during banana fruit ripening and development. Postharvest Biol Technol.

[10] Roy Choudhury S, Roy S, Singh SK, Sengupta DN (2010). Molecular characterization and differential expression of beta-1,3-glucanase during ripening in banana fruit in response to ethylene, auxin, ABA, wounding, cold and light–dark cycles. Plant Cell Rep.

[11] Kesari R, Trivedi PK, Nath P (2007). Ethylene-induced ripening in banana evokes expression of defense and stress related genes in fruit tissue. Postharvest Biol Technol.

[12] Elitzur T, Vrebalov J, Giovannoni JJ, Goldschmidt EE, Friedman H (2010). The regulation of MADS-box gene expression during ripening of banana and their regulatory interaction with ethylene. J Exp Bot.

[13] Yan SC, Chen JY, Yu WM, Kuang JF, Chen WX, Li XP, Lu WJ (2011). Expression of genes associated with ethylene-signalling pathway in harvested banana fruit in response to temperature and 1-MCP treatment. J Sci Food Agric.

[14] Clendennen SK, May GD (1997). Differential gene expression in ripening banana fruit. Plant Physiol.

[15] Gupta SM, Srivastava S, Sane AP, Nath P (2006). Differential expression of genes during banana fruit development, ripening and 1-MCP treatment: Presence of distinct fruit specific, ethylene induced and ethylene repressed expression. Postharvest Biol Technol.

[16] Medina-Suarez R, Manning K, Fletcher J, Aked J, Bird CR, Seymour GB (1997). Gene expression in the pulp of ripening bananas. Two-dimensional sodium dodecyl sulfate-polyacrylamide gel electrophoresis of in vitro translation products and cDNA cloning of 25 different ripening-related mRNAs. Plant Physiol.

[17] Gupta P, Goel R, Pathak S, Srivastava A, Singh SP, Sangwan RS, Asif MH, Trivedi PK (2013). De novo assembly, functional annotation and comparative analysis of Withania somnifera leaf and root transcriptomes to identify putative genes involved in the withanolides biosynthesis. PloS One.

[18] Pathak S, Lakhwani D, Gupta P, Mishra, Brij K, Shukla S, Asif MH, Trivedi PK (2013). Comparative Transcriptome Analysis Using High Papaverine Mutant of Papaver somniferum Reveals Pathway and Uncharacterized Steps of Papaverine Biosynthesis. PloS one.

[19] Blanca J, Esteras C, Ziarsolo P, Pérez D, Fernã Ndez-Pedrosa V, Collado C, Rodrã Guez de Pablos R, Ballester A, Roig C, Cañizares J, Picó B (2012). Transcriptome sequencing for SNP discovery across Cucumis melo. BMC Genomics.

[20] Gonzalez-Ibeas D, Blanca J, Donaire L, Saladie M, Mascarell-Creus A, Cano-Delgado A, Garcia-Mas J, Llave C, Aranda MA (2011). Analysis of the melon (*Cucumis melo*) small RNAome by high-throughput pyrosequencing. BMC Genomics.

[21] Martinelli F, Uratsu SL, Albrecht U, Reagan RL, Phu ML, Britton M, Buffalo V, Fass J, Leicht E, Zhao W, Lin D, D'Souza R, Davis CE, Bowman KD, Dandekar AM (2012). Transcriptome profiling of citrus fruit response to huanglongbing disease. PLoS One.

[22] Yun Z, Jin S, Ding Y, Wang Z, Gao H, Pan Z, Xu J, Cheng Y, Deng X (2012). Comparative transcriptomics and proteomics analysis of citrus fruit, to improve understanding of the effect of low temperature on maintaining fruit quality during lengthy post-harvest storage. J Exp Bot.

[23] Rowland LJ, Alkharouf N, Darwish O, Ogden EL, Polashock JJ, Bassil NV, Main D (2012). Generation and analysis of blueberry transcriptome sequences from leaves, developing fruit, and flower buds from cold acclimation through deacclimation. BMC Plant Biol.

[24] Gongora-Castillo E, Fajardo-Jaime R, Fernandez-Cortes A, Jofre-Garfias AE, Lozoya-Gloria E, Martinez O, Ochoa-Alejo N, Rivera-Bustamante R (2012). The capsicum transcriptome DB: a "hot" tool for genomic research. Bioinformation.

[25] Feng C, Chen M, Xu CJ, Bai L, Yin XR, Li X, Allan AC, Ferguson IB, Chen KS (2012). Transcriptomic analysis of Chinese bayberry (*Myrica rubra*) fruit development and ripening using RNA-Seq. BMC Genomics.

[26] Yu K, Xu Q, Da X, Guo F, Ding Y, Deng X (2012). Transcriptome changes during fruit development and ripening of sweet orange (*Citrus sinensis*). BMC Genomics.

[27] Richardson AC, Boldingh HL, McAtee PA, Gunaseelan K, Luo Z, Atkinson RG, David KM, Burdon JN, Schaffer RJ (2011). Fruit development of the diploid kiwifruit, *Actinidia chinensis* 'Hort16A'. BMC Plant Biol.

[28] Pastore C, Zenoni S, Tornielli GB, Allegro G, Dal Santo S, Valentini G, Intrieri C, Pezzotti M, Filippetti I (2011). Increasing the source/sink ratio in Vitis vinifera (cv Sangiovese) induces extensive transcriptome reprogramming and modifies berry ripening. BMC Genomics.

[29] Guillaumie S, Fouquet R, Kappel C, Camps C, Terrier N, Moncomble D, Dunlevy JD, Davies C, Boss PK, Delrot S (2011). Transcriptional analysis of late ripening stages of grapevine berry. BMC Plant Biol.

[30] Lee JM, Joung JG, McQuinn R, Chung MY, Fei Z, Tieman D, Klee H, Giovannoni J (2012). Combined transcriptome, genetic diversity and metabolite profiling in tomato fruit reveals that the ethylene response factor SlERF6 plays an important role in ripening and carotenoid accumulation. Plant J.

[31] Guo S, Liu J, Zheng Y, Huang M, Zhang H, Gong G, He H, Ren Y, Zhong S, Fei Z, Xu Y (2011). Characterization of transcriptome dynamics during watermelon fruit development: sequencing, assembly, annotation and gene expression profiles. BMC Genomics.

[32] Romero P, Lafuente MT, Rodrigo MJ (2012). The citrus ABA signalosome: identification and transcriptional regulation during sweet orange fruit ripening and leaf dehydration. J Exp Bot.

[33] Kumar S, Asif MH, Chakrabarty D, Tripathi RD, Trivedi PK (2011). Differential expression and alternative splicing of rice sulphate transporter family members regulate sulphur status during plant growth, development and stress conditions. Funct Integr Genomics.

[34] Thatcher SR, Zhou W, Leonard A, Wang BB, Beatty M, Zastrow-Hayes G, Zhao X, Baumgarten A, Li B (2014). Genome-wide analysis of alternative splicing in Zea mays: landscape and genetic regulation. Plant Cell.

[35] Stekel DJ, Git Y, Falciani F (2000). The comparison of gene expression from multiple cDNA libraries. Genome Res.

[36] Brummell DA, Harpster MH (2001). Cell wall metabolism in fruit softening and quality and its manipulation in transgenic plants. Plant Mol Biol.

[37] Munoz-Bertomeu J, Miedes E, Lorences EP (2013). Expression of xyloglucan endotransglucosylase/hydrolase (XTH) genes and XET activity in ethylene treated apple and tomato fruits. J Plant Physiol.

[38] Herrmann KM (1995). The shikimate pathway: early steps in the biosynthesis of aromatic compounds. Plant Physiol.

[39] Boudhrioua N, Giampaoli P, Bonazzi C (2003). Changes in aromatic components of banana during ripening and air-drying. LWT Food Sci Technol.

[40] Nath PTP, Sane VA, Sane AP, NA K (2006). Role of ethylene in fruit ripening. Ethylene Action in Plants.

[41] Hoffmann L, Maury S, Martz F, Geoffroy P, Legrand M (2003). Purification, cloning, and properties of an acyltransferase controlling shikimate and quinate ester intermediates in phenylpropanoid metabolism. J Biol Chem.

[42] Beekwilder J, Alvarez-Huerta M, Neef E, Verstappen FWA, Bouwmeester HJ, Aharoni A (2004). Functional characterization of enzymes forming volatile esters from strawberry and banana. Plant Physiol.

[43] Asif MH, Pathak N, Solomos T, Trivedi PK (2009). Effect of low oxygen, temperature and 1-methylcyclopropene on the expression of genes regulating ethylene biosynthesis and perception during ripening in apple. S Afr J Bot.

[44] Asif MH, Lakhwani D, Pathak S, Bhambhani S, Bag SK, Trivedi PK (2013). Genome-wide identification and expression analysis of the mitogen-activated protein kinase gene family from banana suggest involvement of specific members in different stages of fruit ripening. Funct Integr Genomics.

[45] Jin J, Zhang H, Kong L, Gao G, Luo J (2014). PlantTFDB 3.0: a portal for the functional and evolutionary study of plant transcription factors. Nucleic Acids Res.

[46] Martel C, Vrebalov J, Tafelmeyer P, Giovannoni JJ (2011). The tomato MADS-box transcription factor RIPENING INHIBITOR interacts with promoters involved in numerous ripening processes in a COLORLESS NONRIPENING-dependent manner. Plant Physiol.

[47] Jourda C, Cardi C, Mbeguie AMD, Bocs S, Garsmeur O, D'Hont A, Yahiaoui N (2014). Expansion of banana (*Musa acuminata*) gene families involved in ethylene biosynthesis and signalling after lineage-specific whole-genome duplications. New Phytol.

[48] Asif M, Dhawan P, Nath P (2000). A simple procedure for the isolation of high quality RNA from ripening banana fruit. Plant Mol Biol Rep.

[49] Altschul SF, Gish W, Miller W, Myers EW, Lipman DJ (1990). Basic local alignment search tool. J Mol Biol.

[50] Langmead B, Salzberg S (2012). Fast gapped-read alignment with Bowtie2. Nat Methods.

[51] Anders S, Huber W (2010). Differential expression analysis for sequence count data. Genome Biol.

