# Supplementary material for: Transcriptome analysis of ripe and unripe fruit tissue of banana identifies major metabolic networks involved in fruit ripening process
Source: BMC Plant Biol. 2014 Dec 2;14:316. doi: 10.1186/s12870-014-0316-1 (PMC4263013; doi:10.1186/s12870-014-0316-1)
Supplement: Additional file 3: — Comparative transcripts queried against the TIGR Rice protein database. [file 12870_2014_316_MOESM3_ESM.docx]

**Additional File 11: List of primer sequences of differential genes.**

| **Contig_no./locus Ids** | **Description** | **Forward** | **Reverse** |
| --- | --- | --- | --- |
| contig00961 | ERS1 | TGGCAACGGAAGCTGTAAGT | CTGTCACCTGACCTGCCATT |
| contig06502 | ERS1 | TTCTGTAGCTGCTCAGCCCT | CGAGTCGACTTGAATCCGCT |
| contig00246 | ETR | ACTGAAAACGTAGGGTCGCC | TGTGGCTTACCTACGTGGAG |
| contig02671 | ETR | ATGAAGTCAGGCGTCCACAA | ACGGCGACATATTCCCCAAC |
| contig00120 | CTR1 | GCAAAGCTTGTCTGCACCTGTA | GAGCCTGAACTAGCCTTGAGAG |
| contig03732 | EIN4 | CCTGCAAGGCCAGTCAGAAA | GCCATCACTCTCAGCTAGCAGA |
| contig17908 | EIN4 | AAAGCATGGAAACAGCGCGAAG | CAGCAGGGACCATTCTCTCAC |
| contig18390 | PL1 | TGCTCTTGTCGGACGTCAAC | CATTCATGGATCCACCGCGAT |
| contig07346F | PL2 | TGCAGTCGTGGATGTGGACGC | CATGGTCATCCAGCTGAAGGA |
| contig06876 | PL3 | TCGGCATCGCTTTGAGCTG | CGTCGAGCTACGCGAAGGCT |
| contig08446 | PE1 | GACCACTTTAAAATACGCAGC | GTCGGGAGGGTTATTCAGTACA |
| contig10615 | PE2 | GATGTCCAACGCCCAGACGTG | CGTTGACGAGGGCGAGAGCGT |
| contig03148 | PG1 | GCTCTTCCGGCTACGACTAC | TCCTGATGAAGGTCTCGTGC |
| contig13557 | PG2 | GGCAAGGGACACAACTGGT | AACGTGCTTGCTCCGATGA |
| contig09803 | CELLULASE | CGTCGACACGAACACCATT | GACTCGCACATTCCTCTGCA |
| contig06356 | GT | GAGTGGGGAGATGATGAGGAAC | TTCACGAACGCTACGAACTCAT |
| contig12761 | MT1 | GCAGAGAGACGCTGTGATGAC | CCTCTGCCGGAAGCCTCTCGT |
| contig17111 | MT2 | GAGTAACATAACGTCAGCCG | CCGCACATGAAGTGCACCGT |
| contig17350 | MT3 | TATCAGACTTGCTGCCTTCGT | TCCTGCACGACTGGAAAGATG |
| GSMUA_Achr2T16160_001 | ACT101 | ATGACATGGAAAAGATCTGGCA | CCTGAATGGCAACATACATAGC |
