# Supplementary material for: Transcriptome analysis of ripe and unripe fruit tissue of banana identifies major metabolic networks involved in fruit ripening process
Source: BMC Plant Biol. 2014 Dec 2;14:316. doi: 10.1186/s12870-014-0316-1 (PMC4263013; doi:10.1186/s12870-014-0316-1)
Supplement: Additional file 10: — Description of genes associated with identified SSRs. [file 12870_2014_316_MOESM10_ESM.ppt]

## Slide 1
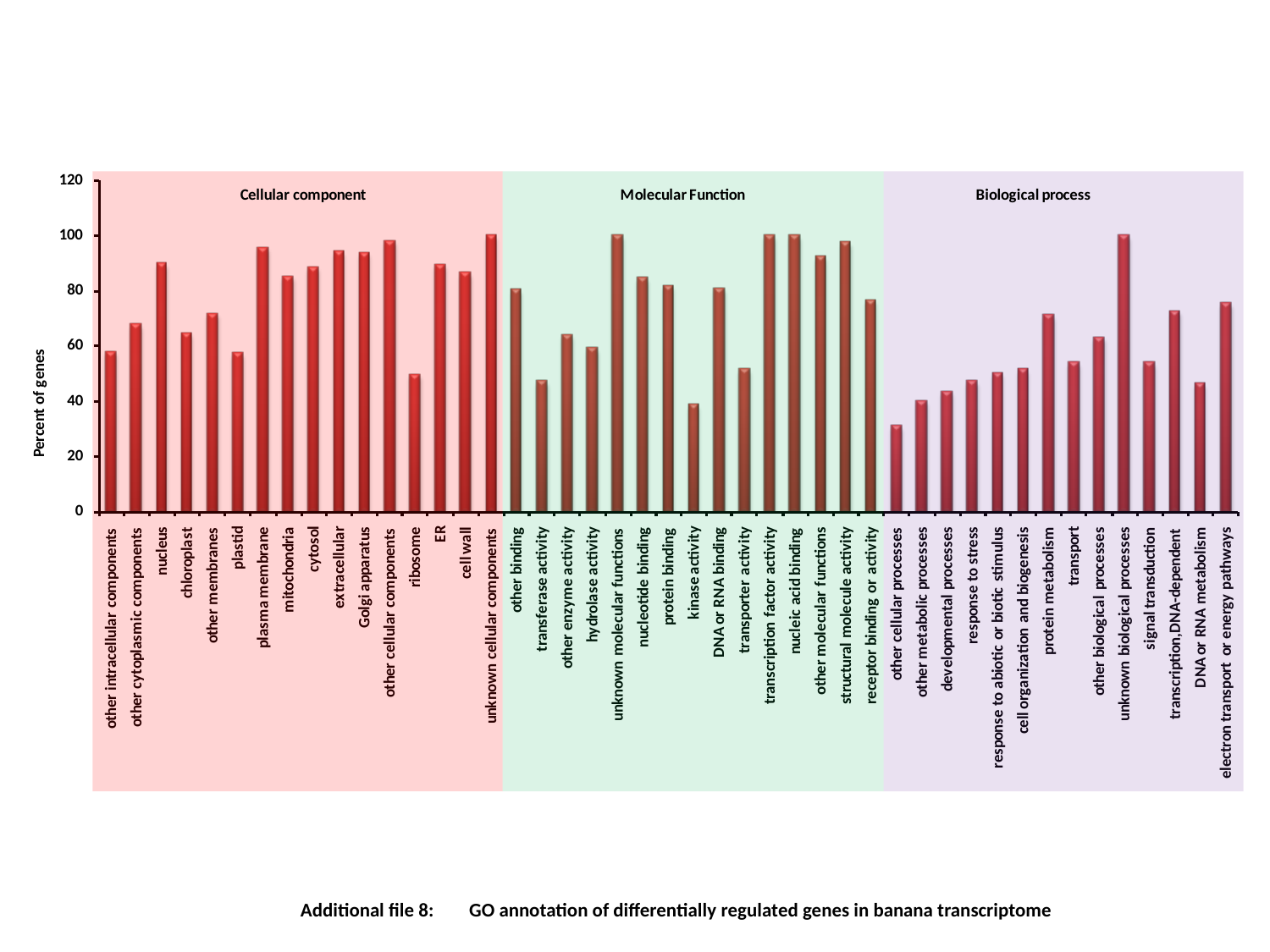

| Additional file 8: GO annotation of differentially regulated genes in banana transcriptome |
| --- |
